# On our own feet: preparing for the donor to leave

**Published:** 2013

**Authors:** Bo Wiafe

**Affiliations:** Regional Director for Africa: Operation Eyesight Universal, Accra, Ghana. **bwiafe@operationeyesight.com**

**Figure:**
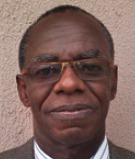
Bo Wiafe

Many eye care programmes are supported by donors and once this funding stops, they can collapse. In this article, I will explain how some programmes and institutions have been able to keep going despite losing their donor funding. I have had the opportunity to work with government, church-run and privately owned institutions in Ghana, Zambia, Kenya and Rwanda, and will share with you what we have done to promote sustainability in some of these organisations.

## Starting the thinking process

With any programme, however secure the funding might seem, it is helpful to think about how long funding might continue and plan accordingly. In general, as mentioned on pages 41–43, it is best to use external funding for development and start-up costs (training, equipment, infrastructure) and then work towards sustainability, meeting the ongoing or running costs from other sources that are reliable in the long term.

The programmes and institutions with which we worked had mostly been provided with infrastructure, equipment, staff training and, in some instances, some consumables, to start their work.

Money was needed for the following ongoing costs:

- salaries (40–70% of total ongoing costs)
- consumables
- communication (internet, telephone, publicity and awareness creation)
- office and other costs (including water, electricity, maintenance, etc.).

As a next step, we acknowledged that tensions existed between sustainability on the one hand, and creating quality services that everyone could access at an affordable price on the other.^1^ In particular, we acknowledged that income might be too low to meet operating costs, while staying true to the principles of quality, equal access and affordability.

## Reducing costs

The two key principles we applied were:

1. To keep costs as low as possible.
2. Where costs could not be reduced further, to make the best use of each resource.

### Personnel

Only essential personnel were recruited. A typical team would consist of one ophthalmologist, two optometrists, one administrator, two ophthalmic nurses, two ophthalmic technicians, five nurse assistants, one equipment technician, two housekeepers, and one driver. The number of staff can be increased as the workload increases.

We reviewed the effectiveness of all members of the team every year and determined whether there was a way to reduce the costs associated with their work or to improve their output (e.g. the number of patients seen or treated).

‘However secure the funding might seem, it is helpful to think about how long funding might continue and plan accordingly’

### Utilities (water, electricity, phones)

In a facility, much power and water is wasted because staff are unaware of the impact it can have on expenditure. We drew their attention to this and encouraged them to make sure lights and water were turned off at the end of the day. Any leaks and faults were reported and fixed immediately.

#### Use of official vehicles

Strict control measures were applied, including the use of a log book. The cost of running hospital vehicles was reduced by half. The log book also helped each hospital to know which department to charge for each trip.

#### Bulk buying

Hospitals were encouraged to change from purchasing on a monthly basis to annual or quarterly bulk purchasing of drugs and consumables, as well as quarterly purchasing of spectacle frames.

#### Non-eye care services

Security, grounds maintenance and catering are not part of the core business of providing eye care, and it may be cheaper to enter into contracts with external companies to provide these services (known as outsourcing). We considered each case carefully to ensure that it made financial sense to outsource, and to ensure that quality would be maintained at an acceptable level.

### Increasing income

We increased the number of appointments and operations by attracting more patients and working more effectively.

We also diversified our sources of funding by linking up with health insurance schemes, for example by ensuring that each eye clinic or department was linked with (or accredited by) at least one local insurance scheme.

**Figure:**
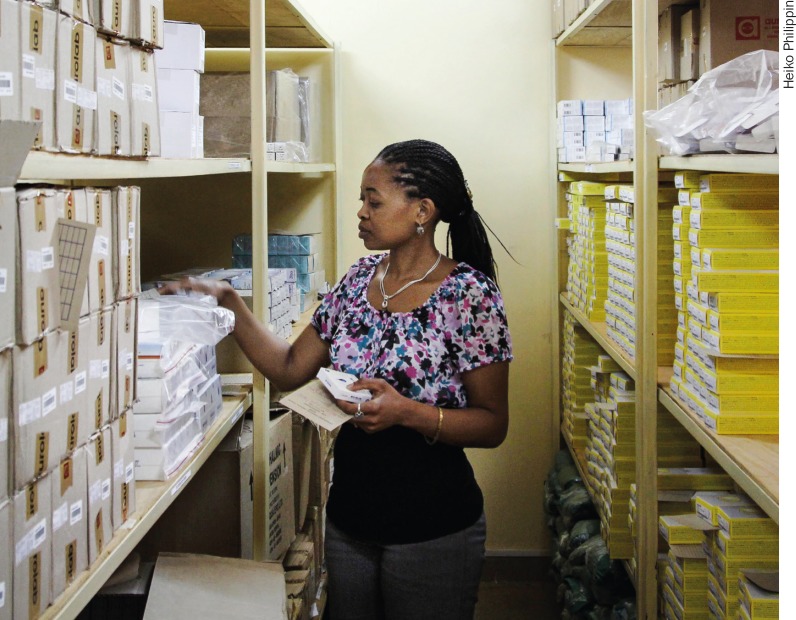
It is cheaper to buy drugs and consumables in bulk.

Accreditation can be a lengthy process but ultimately it is worthwhile. It generally involves:

- ensuring that the facility is certified by the appropriate authority (e.g. the ministry of health or the district health authority)
- applying to the relevant insurance scheme(s)
- completion of the accreditation forms
- inspection by the insurance scheme
- receiving certification.

Government insurance schemes (or national health insurance schemes) have helped to increase the numbers of patients who use the facilities. This is because patients who would otherwise delay coming to the hospital came more readily because they did not have to pay cash for services; these costs were covered by the insurance scheme.

#### Attracting more patients

We decided to attract all kinds of patients to the clinics by creating a one-stop facility, like a supermarket, with a comprehensive list of eye services, including:

- eye surgery
- eye tests
- spectacle dispensing.

In addition, we visited companies and factories to provide screening services for workers who are too busy to come to the clinics.

### Keeping quality high

The quality of services can be kept high by applying a number of strategies:

1. **Human resources.** Select staff carefully and treat them well so they always want to give their best. This includes providing staff with the equipment they need to do their work, offering training programmes to build capacity, and providing a safe working environment.
2. **Equipment.** Use initial or start-up funding to equip facilities with the equipment needed to make accurate diagnoses. Employ a part-time equipment maintenance officer, or assign equipment maintenance responsibilities to another staff member (while ensuring they are properly trained and resourced). This will ensure that equipment is properly maintained, which means it will remain in good working condition for as long as possible.
  **Standardisation.** Standardising procedures/protocols and equipment improves efficiency and enhances the quality of the outcomes.
3. **Monitoring.** Put systems in place for monitoring patient outcomes and patient satisfaction, and take steps to make any changes needed.

### Challenges

It has not been easy to get to where we are without challenges. In the case of the government institutions, one of the main challenges is to encourage administrators to think of creative solutions. Other challenges are listed below.

- Putting funds into a single account, also known as commingling of funds (i.e. not separating them into different accounts). This makes tracking progress and reporting to sponsors difficult; for example, funds budgeted for one activity may end up in expenditures for another (see article on page 48 for a suggested way to handle this problem).
- Delays in the payments by the National Health Insurance Scheme to the hospitals.
- Difficulties in retaining staff, especially in government institutions.
