# Supplementary material for: On our own feet: preparing for the donor to leave
Source: Community Eye Health. 2013;26(83):45–6. (PMC3864052)
Supplement: Related texts

# Brief report: National health insurance schemes in Ghana

**Published:** 2013

National health insurance came into being in Ghana with the passing of the National Health Insurance Act in 2003.

Its three main sources of funding are:

- the National Health Insurance Levy, a 2.5% value-added tax on goods and services (70%)
- social security taxes, paid by people in formal employment (23%)
- individual premiums, paid by insurance scheme members (5%).

The implementation of national health insurance has increased access to public health care services and raised public expectations: there is evidence that enrolled individuals are more likely to seek care for illness or injury.^1^, ^2^ This translates to greater patient numbers, which improves sustainability.

Once a health care facility has been accredited (registered and approved) by the National Health Insurance Authority, it is permitted to display the sign of the relevant insurance scheme(s). (There are three different types of insurance schemes permitted by the act, and 145 are currently registered.)

Members of the insurance scheme can access services at accredited institutions without having to pay at the point of service; the scheme then reimburses the health care facility according to a set tariff. Everyone has to pay a registration fee to obtain an insurance card, but some people are exempt from paying the premiums, including people over 70, pregnant women, children (if both their parents enroll) and the very poor.

Eye care benefits include cataract and eyelid surgery, biometry, assessment of visual fields, refraction, and basic ophthalmic preparations, but not spectacles and other optical devices.

**Figure:** Eye care providers have to be accredited by the National Health Insurance Authority before they can display the sign of the relevant scheme at their institution.

## References

[1] WitterSGarshongB Something old or something new? Social health insurance in Ghana. BMC Int Health Hum Rights 2009; 9: 201971558310.1186/1472-698X-9-20PMC2739838

[2] BlanchettNJFinkGOsei-AkotoI Ghana Med J. 2012;46(2):76-8422942455PMC3426378
